# TRIM14 expression is regulated by IRF‐1 and IRF‐2

**DOI:** 10.1002/2211-5463.12682

**Published:** 2019-07-01

**Authors:** Jingang Cui, Xiao Xu, Yutong Li, Xiaomei Hu, Yingpeng Xie, Juan Tan, Wentao Qiao

**Affiliations:** ^1^ Key Laboratory of Molecular Microbiology and Technology Ministry of Education College of Life Sciences Nankai University Tianjin China; ^2^ Present address: Department of Clinical Laboratory The First Affiliated Hospital of Zhengzhou University Zhengzhou 450052 China

**Keywords:** IRF‐1, IRF‐2, ISRE, promoter, TRIM14

## Abstract

Tripartite motif‐containing 14 (TRIM14) is a mitochondrial adaptor that promotes innate immune signaling and plays important roles in antiviral defense. Expression of TRIM14 is induced by interferon (IFN)‐I. However, the mechanism by which IFN‐I induces TRIM14 production is not yet determined. In this study, we have examined the function of *TRIM14* promoter and found that a GC box and an IFN‐stimulated response element (ISRE) are necessary for the basal level transcription of *TRIM14*. We further observed that IFN‐I activates the *TRIM14* promoter through the ISRE. In particular, interferon regulatory factor (IRF)‐1 and IRF‐2 bind to the *TRIM14* promoter and activate transcription of *TRIM14*. Moreover, knockdown of IRF‐1 reduces the stimulation of *TRIM14* transcription by IFN‐α, suggesting that IRF‐1 is involved in the activation of *TRIM14* by IFN‐I. IRF‐2 has little effect on IFN‐α‐induced *TRIM14* transcription but is essential for the basal transcription of *TRIM14*.

**Abbreviations**
IFN: interferon
IRF: interferon regulatory factor
ISG: interferon‐stimulated gene
ISRE: IFN‐stimulated response element
SD: standard deviation
STAT: signal transducer and activator of transcription
TRIM14: tripartite motif‐containing 14
WT: wild‐type

Tripartite motif (TRIM) proteins contain a RING finger, one or two B‐box motifs and a coiled‐coil motif. Ample studies have demonstrated that TRIM family proteins play important roles in antiviral innate immune response. For example, TRIM22 activates nuclear factor‐κB signaling 1, and TRIM56 promotes double‐stranded DNA‐stimulated interferon induction by ubiquitination of stimulator of interferon gene (STING) 2, 3.

Tripartite motif‐containing 14 (TRIM14) is a member of the tripartite‐motif protein family; it is expressed in a variety of tissues 4. As a regulatory factor, it plays an important role in the innate immune response. In the retinoic acid‐inducible gene I (a sensor of virus double‐stranded RNA) signaling pathway, TRIM14 acts as a link protein in activating the expression of interferon (IFN)‐I. TRIM14 also enhances the IFN‐I signaling pathway by stabilizing cyclic GMP–AMP synthase (a DNA virus sensor). IFN‐I in turn upregulates the expression of TRIM14, thus enhancing the innate immune response mediated by the virus 5, 6. In addition, TRIM14 is necessary for retinoic acid‐inducible gene I‐mediated innate antiviral immunity by forming a WHIP–TRIM14–PPP6C mitochondrial complex 7.

Interferons regulate downstream genes through the Janus kinase–signal transducer and activator of transcription (STAT) pathway 8. IFN‐I binds to the interferon α and β receptor and activates the Janus kinase–STAT pathway to enhance the transcription of interferon‐stimulated genes (ISGs) 9, 10, 11. IFN regulatory factors (IRFs) are also involved in IFN‐mediated signaling pathways. The IRF family consists of nine members (IRF‐1–IRF‐9), which were characterized as transcriptional regulators of IFN and IFN‐inducible genes 12. IRF‐1 was the first IRF identified and is characterized by its ability to bind to the IFN‐β promoter and activate IFN‐β expression 13. IRF‐1 can regulate some IFN‐regulated genes by directly binding to the IFN‐stimulated response element (ISRE) of their promoters, including RANTES/CC15 14 and low molecular weight protein 7 15. IRF‐2 has a similar structure to IRF‐1; it is thus considered as a transcriptional inhibitor of IRF‐1‐mediated transcriptional activation. However, IRF‐2 also has a transcriptional activation function; for example, it can activate the expression of histone H4 16.

Many TRIM genes are regulated by IFN‐I or IFN‐II 17, 18, and IFN‐I can induce the expression of TRIM14. However, it has not been determined which elements and transcription factors activate TRIM14 expression following IFN‐I induction. In this study, we have determined that the ISRE in the *TRIM14* promoter is necessary for the regulation of *TRIM14* by IFN‐I. Furthermore, we found that IRF‐1 and IRF‐2 bind to the ISRE and mediate transcriptional activation of *TRIM14* at different stages. IRF‐1 is involved in the activation of *TRIM14* by IFN‐I, whereas IRF‐2 is essential for the basal transcription of *TRIM14*.

## Results

### Promoter activity analysis of *TRIM14*

**Figure 1:**
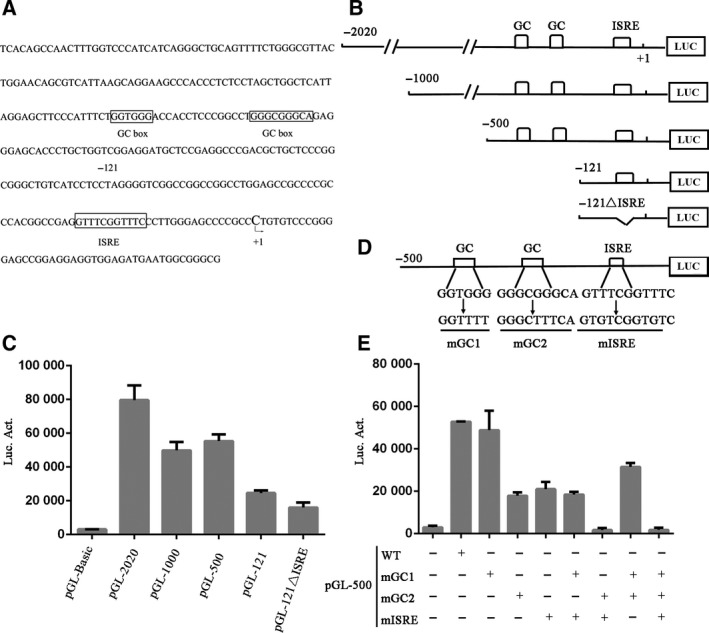
Identification and analysis of *TRIM14* promoter. (A) Arrow indicates the starting point of transcription, and the C is designed as +1. The potential *cis*‐elements are boxed. (B) The 5′‐truncated plasmids of *TRIM14* promoter. (C) The promoter constructs were transfected into HeLa cells. After transfection for 48 h, a luciferase assay was performed and β‐gal activity was used as a normalization control for the luciferase activity. (D) *TRIM14* promoter core region mutation in pGL‐500. (E) The wild‐type (WT) or mutant PGL‐500 was transfected into HeLa cells; luciferase was detected 48 h after transfection. Results are presented as mean ± standard deviation (SD), and data used for the analysis were from three independent experiments.

In order to understand the mechanism by which *TRIM14* expression is induced by IFNs, we analyzed its promoter activity. We determined the transcriptional initiation site of *TRIM14* through 5′‐rapid amplification of cDNA ends (5′‐RACE) PCR. The products were cloned into pMD18‐T and sequenced. Sequencing results show that seven clones contain two transcriptional start sites and five of these clones started with cytosine, and we thus defined this cytosine as the transcription start site and mark it as +1 (Fig. 1A). Then, we cloned the 2020 bp DNA upstream of the transcription initiation site and performed sequence analysis. The results show that it contains three potential *cis*‐acting components, including two GC boxes (GC box 1 and GC box 2) and one ISRE (Fig. 1A).

To verify whether the predicted *cis*‐acting elements are involved in regulating *TRIM14* promoter activity, we constructed a series of 5′ truncated plasmids (Fig. 1B), and then transfected these reporter plasmids into HeLa cells and measured the luciferase activity 48 h post‐transfection. The basal transcriptional activity of truncated *TRIM14* promoter from −500 to −121 decreased significantly and the basal transcriptional activity of the *TRIM14* promoter was further reduced by further truncations (Fig. 1C). The −500 to +1 bp region is consistent with our predicted positions of the *cis*‐acting elements. In order to further confirm whether the two GC boxes and the ISRE are involved in regulating the basic transcriptional activity of the *TRIM14* promoter, we constructed a series of mutations on pGL‐500 (Fig. 1D). As shown in Fig. 1E, the basal transcriptional activity decreased by 75% (GC2 mutation) and 70% (ISRE mutation). However, GC1 mutation does not affect the basal transcriptional activity of the *TRIM14* promoter (Fig. 1E, column 3, 7, 9). Therefore, we conclude that GC2 and ISRE are essential elements in the basal transcription of TRIM14.

### The ISRE is essential for IFNs to activate *TRIM14* promoter

**Figure 2:**
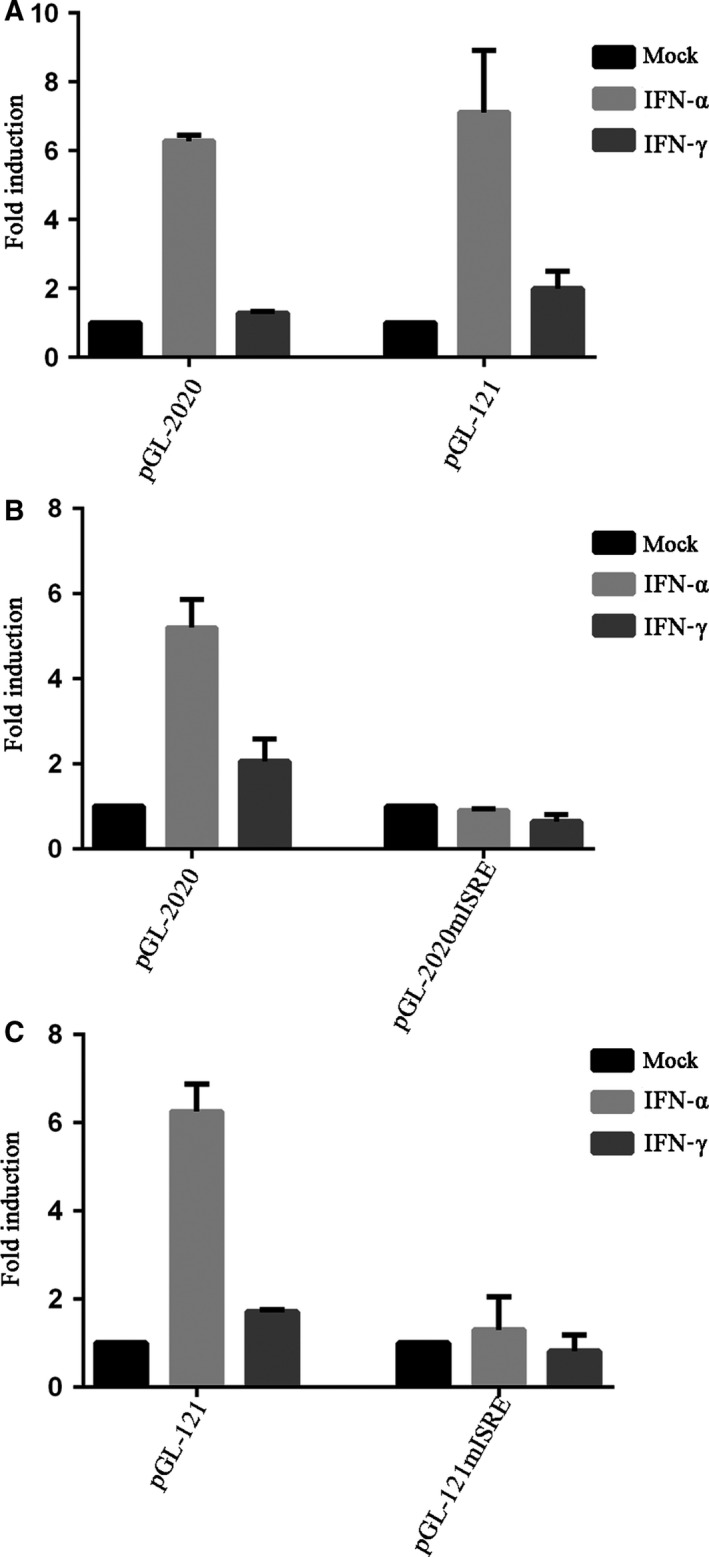
IFNs enhance *TRIM14* transcription through ISRE. (A) pGL‐2020 or pGL‐121 was transfected into HeLa cells. After 32 h of transfection, the cells were treated with IFN‐α or IFN‐γ (10 ng·mL^−1^) for 16 h. Luciferase activity was determined after 48 h transfection. (B, C) pGL‐2020 or pGL‐2020ISRE or pGL‐121 or pGL‐121mISRE was transfected into HeLa cells and HeLa cells were stimulated with IFN‐α or IFN‐γ (10 ng·mL^−1^) 16 h before luciferase detection. Results are presented as mean ± SD, and data used for the analysis were from three independent experiments.

To verify whether the *TRIM14* promoter is regulated by IFNs, HeLa cells were transfected with pGL‐2020 or pGL‐121. Thirty‐two hours post‐transfection, cells were treated with IFN‐α or IFN‐γ for 16 h. As shown in Fig. 2A, both IFN‐α and IFN‐γ can upregulate gene expression from the *TRIM14* promoter, and IFN‐α is more potent than IFN‐γ. pGL‐2020 and pGL‐121 are upregulated to almost the same extent by IFNs, indicating that only −121 to +1 bp is the region that responds to IFN activation. The region −121 to +1 contains an ISRE. To further confirm whether IFNs activate the promoter via the ISRE, we constructed two ISRE mutation plasmids (pGL‐2020mISRE, pGL‐121mISRE). We found that the *TRIM14* promoter does not respond to IFNs when the ISRE is mutated (Fig. 2B,C). These results demonstrate that the ISRE is essential for IFNs to activate the *TRIM14* promoter.

### IRF‐1 and IRF‐2 bind to the ISRE to increase *TRIM14* expression

**Figure 3:**
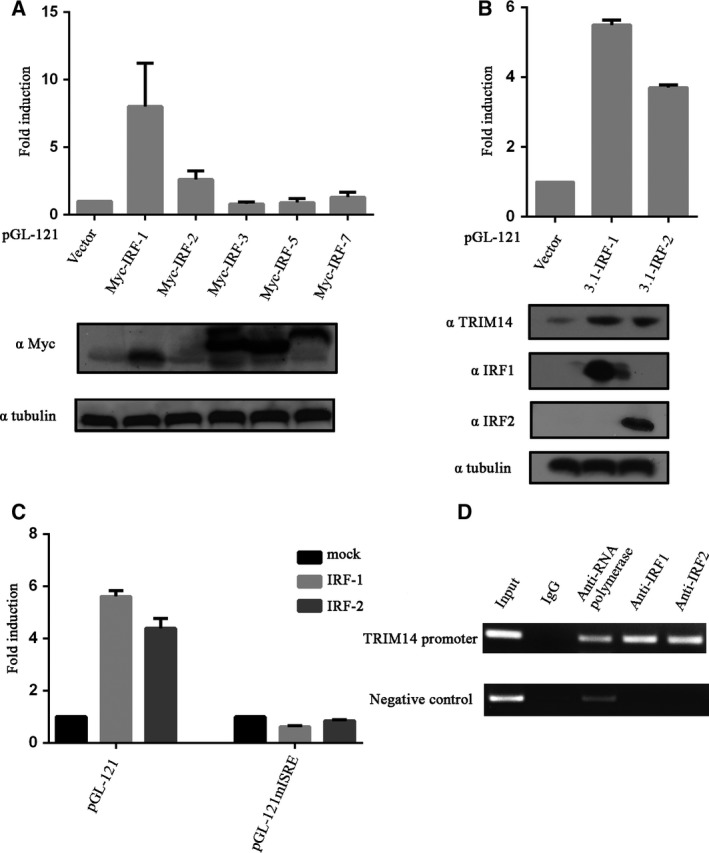
IRF‐1 and IRF‐2 bind to the ISRE to increase *TRIM14* expression. (A, B) pGL‐121 with IRFs transfected into HeLa cells; luciferase activity was measured after transfection for 48 h and western blot analysis was performed. (C) PGL‐121 or PGL‐121mISRE was co‐transfected with pcDNA3.1 or pcDNA3.1‐IRF‐1 or pcDNA3.1‐IRF‐2 into HeLa cells. Luciferase activity was measured 48 h after transfection. (D) HeLa cells were treated with IFN‐α (10 ng·mL^−1^) for 12 h and detected with anti‐IRF‐1, anti‐IRF‐2 or control IgG and anti‐RNA polymerase. The designed *TRIM14* ISRE primer was used to amplify precipitated DNA by PCR. Results are presented as mean ± SD, and data used for the analysis were from three independent experiments.

Many members of the IRF family can regulate transcription of IFN‐stimulated genes. To determine whether the IRF family is involved in regulating *TRIM14*, we co‐transfected Myc–IRF‐1, Myc–IRF‐2, Myc–IRF‐3, Myc–IRF‐5 and Myc–IRF‐7 encoding plasmids with pGL‐121 into HeLa cells and examined pGL‐121 promoter activity. As shown in Fig. 3A, overexpression of IRF‐1 and IRF‐2 can significantly activate the *TRIM14* promoter, and the activation by IRF‐1 is greater than that by IRF‐2. Because Myc‐IRF‐2 plasmid did not express (Fig. 3A), we co‐transfected pGL‐121 with pCDNA3.1‐IRF‐1 or pCDNA3.1‐IRF‐2 (expressed well) into HeLa cells and pGL‐121 promoter activity was examined. The results showed that IRF‐1 and IRF‐2 activated the *TRIM14* promoter and increased TRIM14 protein expression (Fig. 3B).

ISRE is a known binding site for many IRFs. To demonstrate whether IRF‐1 and IRF‐2 regulate *TRIM14* transcription by binding to the ISRE in the *TRIM14* promoter, we co‐transfected IRF‐1 or IRF‐2 with pGL‐121 or pGL‐121mISRE into HeLa cells. The results showed that IRF‐1 and IRF‐2 did not activate the pGL‐121mISRE (Fig. 3C), indicating that IRF‐1 and IRF‐2 activate *TRIM14* transcription via the ISRE. To further demonstrate whether IRF‐1 and IRF‐2 both bind to the ISRE and regulate the *TRIM14* promoter, we performed a ChIP assay to determine whether endogenous IRF‐1 and IRF‐2 can bind to ISRE with IFN‐α stimulation. The PCR primers cover the region from −150 to +1, which only contains the ISRE. The results showed that endogenous IRF‐1 and IRF‐2 bound to the ISRE (Fig. 3D), suggesting that IRF‐1 and IRF‐2 regulate *TRIM14* expression by binding to the ISRE.

### IRF‐1 and IRF‐2 differentially upregulate *TRIM14* expression with IFN‐α treatment

**Figure 4:**
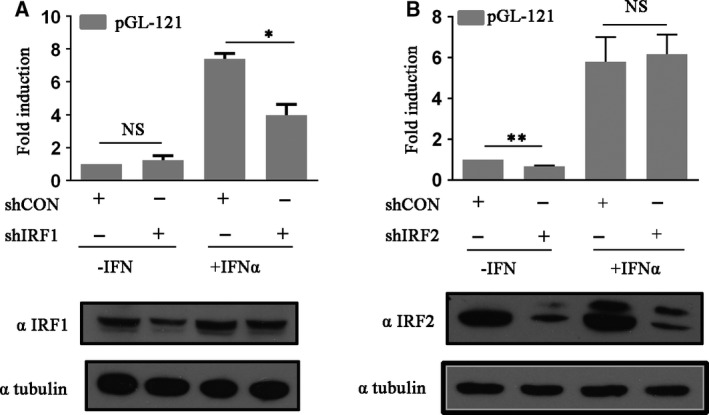
IRF‐1 and IRF‐2 differentially upregulate *TRIM14* expression on IFN‐α treatment. (A, B) shcontrol, shIRF‐1 or shIRF‐2 co‐transfected HeLa cells with pGL‐121. Cells were treated with or without IFN‐α (10 ng·mL^−1^) for 16 h; luciferase activity was measured and western blot analysis was performed. Results are presented as mean ± SD, and data used for the analysis were from three independent experiments. Values are the mean and standard error of three independent experiments, *P < 0.05, **P < 0.01.

To determine whether IRF‐1 and IRF‐2 are necessary for IFN‐α‐induced *TRIM14* expression, we knocked down IRF‐1 and IRF‐2 by shIRF‐1 and shIRF‐2 in HeLa cells. We co‐transfected shIRF‐1 or shIRF‐2 and pGL‐121 into HeLa cells. pGL‐121 transcriptional activity and levels of endogenous IRF‐1 and IRF‐2 were measured. As shown in Fig. 4A, when the endogenous IRF‐1 in HeLa cells was knocked down, basal transcriptional activity of *TRIM14* decreased moderately, but transcriptional activity decreased by 47% after IFN‐α stimulation. In contrast, knockdown of IRF‐2 resulted in the suppression of basal *TRIM14* expression, but did not affect the IFN‐inducible expression (Fig. 4B). Overall, these data indicate that IRF‐1, but not IRF‐2, is involved in the activation of *TRIM14* by IFN‐α.

## Discussion

Interferons play an important role in the antiviral innate immune response. IFNs can activate the expression of ISGs, which inhibit viruses at the different replication stages. TRIM14 is an ISG and antagonizes a variety of viruses, including mouse leukemia virus, sindbis virus, hepatitis C virus and others 19, 20, 21. TRIM14 can be induced by IFN‐I and enhances the host's immune response to virus infection 5, 6. In this study, we determined the specific mechanism of IFN‐I activation of TRIM14 expression.

First, we cloned and analyzed the features of the *TRIM14* promoter. The *TRIM14* promoter has a GC box and an ISRE, but no TATA box, and transcription initiates at two start sites. Base composition analysis showed that the GC content near the initiator is 60.2% (−500 to +1). There are two typical promoter types in the mammalian genome, which are classified mainly by GC content and CpG dinucleotide frequency 22. The high CG promoters (high GC content and high CpG dinucleotide frequency) typically contain multiple transcription start sites and do not contain a TATA box, and genes with high CG promoters are widely expressed throughout the biological cycle. The low CG promoters (low GC content and low CpG dinucleotide frequency) contain a single transcriptional start site and TATA‐box enrichment; low CG promoters are associated with tissue‐specific expression of genes 23. Based on the features of the *TRIM14* promoter, it belongs to the high CG promoters. This is also consistent with its expression profiles in many tissues 4.

ISREs control the expression of many ISGs. Similar to some IFN‐induced genes, such as *BAFF*,* TRIM21* and *TLR3* 24, 25, 26, the promoter of *TRIM14* contains an ISRE located from −27 to −17. We determined that IFN‐I activates *TRIM14* transcription through the ISRE. ISREs are recognized by different IRFs, and IRF‐1 can enhance transcription of many ISGs through an ISRE 27, such as ISG20 and interleukin‐7 28, 29. IRF‐2 is also known to recognize ISREs 30, and IRF‐2 is considered as a transcription inhibitor and antagonist of IRF‐1 31, 32. In this study, we observed that IRF‐1 and IRF‐2 bound to the ISRE and both activated the transcription of *TRIM14*, although the activation by IRF‐1 is stronger than that of IRF‐2. Our data also show that IRF‐1 and IRF‐2 differentially upregulate *TRIM14* expression upon IFN‐I treatment. IRF‐2 maintains a basal level expression and IRF‐1 is involved in the IFN‐I‐inducible expression of *TRIM14*. In addition to regulating the transcription of *TRIM21*,* TLR3*,* IFITM3* and other ISGs by IRF‐1 and IRF‐2 33, our data support further that IRFs play a crucial role in coordinating the transcriptional activation in the cellular antiviral response.

**Figure 5:**
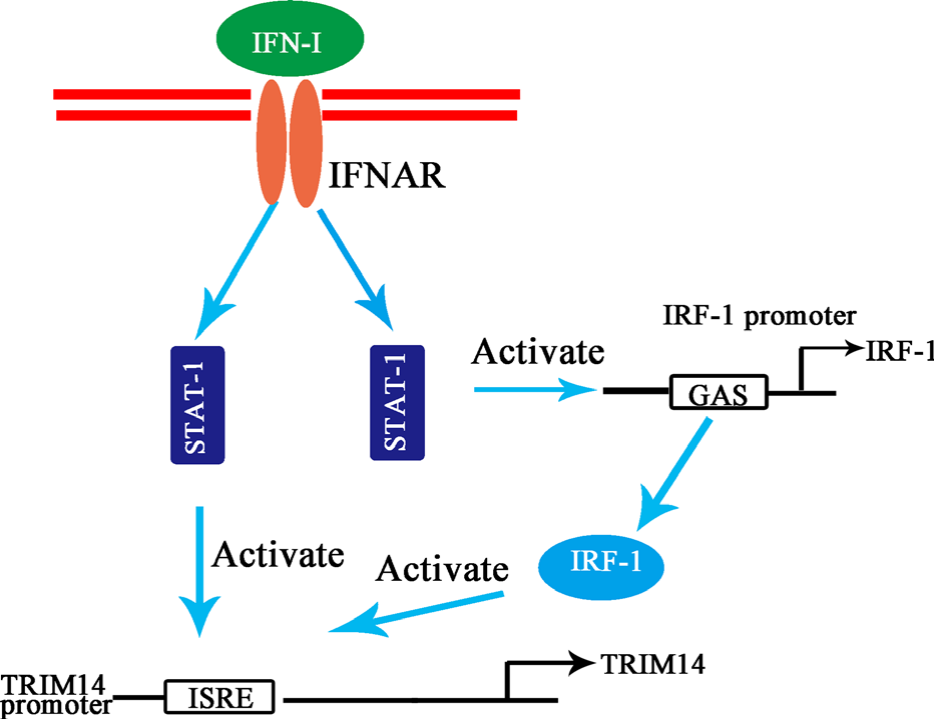
Model of regulation of *TRIM14* transcription by IFN‐I. GAS, gamma activation sequence; IFNAR, Interferon‐alpha/beta receptor.

Previous studies have shown that STAT‐1 is essential for IFN‐I induction of TRIM14 34. They found that TRIM14 was not induced by IFN‐α in STAT‐1 knockout cells, while our work showed that knocking down IRF‐1 also reduce the IFN‐α activation of *TRIM14*. Considering that STAT‐1 can upregulate IRF‐1 and enhance IRF‐1 translocation into the nucleus and binding to ISRE 35, we propose that IRF‐1 enhances IFN activation of *TRIM14* through STAT‐1, creating a positive feedback effect. IRF‐1 is an auxiliary amplifier of IFN‐I activation of *TRIM14*. This model is illustrated in Fig 5.

In conclusion, our study demonstrates that IFN‐I upregulates TRIM14 expression by recruiting IRF‐1 to the ISRE of the *TRIM14* promoter. Our findings on the specific regulation mechanism of TRIM14 expression not only advance our understanding of the physiological function of TRIM14, but also elucidate how TRIM14 is regulated in the innate immune response.

## Materials and methods

### Constructs and antibodies

Plasmids pGL‐2020, pGL‐1000, pGL‐500, pGL‐121 and pGL‐121∆ISRE were constructed by cloning the PCR‐amplified fragment of the *TRIM14* promoter into pGL3‐basic (Promega, Madison, WI, USA), and the primers are listed in Table 1. pGL‐500 mutants were constructed using a site‐directed mutagenesis kit (Toyobo, Osaka, Japan), and the primers used are listed in Table 1. Plasmids IRF‐1, IRF‐2, IRF‐3, IRF‐5 and IRF‐7 were constructed by inserting the coding sequence into the pCDNA3.1 (+) (Thermo Fisher Scientific, Waltham, MA, USA) or pCMV‐Tag3B (Agilent, Santa Clara, CA, USA) vector. The shRNA constructs for IRF‐1 and IRF‐2 were constructed using the pSIREN‐RetroQ vector (Clontech, Mountain View, CA, USA). The target sequence for IRF‐1 was: 5′‐GGGGTACCTACTCAATGAACCT‐3′; and for IRF‐2: 5′‐GGGGTACCTACTCAATG‐AACCT. Sequences of all of the constructs were confirmed by sequencing. Antibodies against IRF‐1, IRF‐2, Myc and Flag were purchased from Santa Cruz Biotechnology (Dallas, TX, USA), α‐tubulin from Sigma‐Aldrich (St. Louis, MO, USA), and TRIM14 antibody from Abcam (Cambridge, UK).

**Table 1:** Primers for amplifying *TRIM14* promoter fragments and site‐directed mutagenesis.

Primer	Sequence (5′–3′)
P2020	CGACGCGTCCACCTTAACGCTACAATAATTGTGCTTCC
p1000	GACGCGTCTGCAACCTTGCACAAAGAACC
P500	GACGCGTGTTTTGCCTTTAAAAGCC
P121	GACGCGTGAGGCCCGACGCTGCTCCCG
Plow	CAGATCTCGCCATTCATCTCCACCTCCTCC
P121∆ISRE‐F	GACGCGTGAGGCCCGACGCTGCTCCCG
P121∆ISRE‐R	CAGATCTCGCCATTCATCTCCACCTCCTCCGGCTCCCCGGGACACAGGGCGGGGCTCCCAAGGCTCGGCCGTGGGCGGGGCGGCTCC
mGC1‐F	CTTCCCATTTCTGGTTTTACCACCTCCCGGCC
mGC1‐R	GGCCGGGAGGTGGTAAAACCAGAAATGGGAAG
mGC2‐F	CCTCCCGGCCTGGGCTTTCAGAGGGAGCACCCTG
mGC2‐R	CAGGGTGCTCCCTCTGAAAGCCCAGGCCGGGAGG
mISRE‐F	ACGGCCGAGGTGTCGGTGTCCCTTGGGAGCCCCGC
mISRE‐R	TCCCAAGGGACACCGACACCTCGGCCGTGGGCG

### Cell culture and transfection

HeLa cells (maintained in our lab) were grown in Dulbecco's modified Eagle's medium (Gibco, Gaithersburg, MD, USA) medium supplemented with 10% FBS (BI) in 5% CO_2_ at 37°. Transfection was performed using polyetherimide reagent (Sigma‐Aldrich).

### 5′‐Rapid amplification of cDNA ends

5′‐Rapid amplification of cDNA ends was used to characterize the 5′‐end of *TRIM14* transcript using Firstchoice RLM‐RACE Kit (Ambion, Austin, TX, USA). Total RNA was extracted from HeLa cells (Thermo Fisher Scientific). DNA fragments were amplified by nested PCR. The sequence of the first round of PCR specific primer was 5′‐TGCCAGCTGCTTTAAACATTC‐3′, and primer used in the second round was 5′‐GCGGCAGCGGCGACAGAAG‐3′. PCR products were cloned into the pMD18‐T vector for further analysis.

### Chromatin immunoprecipitation

ChIP analysis was conducted based on the manufacturer's instructions using an EZ‐chip kit (Millipore, ‎Burlington, MA, USA). PCR primers used for amplifying the *TRIM14* promoter are as follows: forward, 5′‐GAGGCCCGACGCTGCTCCCG‐3′ and reverse: 5′‐CGCCATTCATCTCCACCCCTCC.

### Luciferase reporter assay

Luciferase plasmids and β‐galactosidase expression plasmids were transfected into cells. Luciferase activity was determined with a luciferase report system (Promega) and normalized to β‐galactosidase activity.

## Conflict of interest

The authors declare no conflict of interest.

## Author contributions

JC, JT and WQ participated in the design of the study; JC, XX, YL, XH and YX helped in data collection and the interpretation of data. JC drafted the article. JT and WQ revised the article and gave final approval of the version to be submitted.
